# Dentist Related Factors Associated with Implementation of COVID-19 Protective Measures: A National Survey

**DOI:** 10.3390/ijerph18168381

**Published:** 2021-08-08

**Authors:** Joana Christina Carvalho, Dominique Declerck, Wolfgang Jacquet, Peter Bottenberg

**Affiliations:** 1 Faculty of Medicine and Dentistry, UCLouvain, 1200 Brussels, Belgium; 2 Department of Oral Health Sciences, Population Studies in Oral Health, KU Leuven, 3000 Leuven, Belgium; dominique.declerck@uzleuven.be; 3 Oral Health Research Cluster, Faculty of Medicine and Pharmacy, VUB, 1090 Brussels, Belgium; wolfgang.jacquet@vub.be (W.J.); peter.bottenberg@vub.be (P.B.)

**Keywords:** corona virus, COVID-19, severe acute respiratory syndrome corona virus type 2, online survey, dental care, infection control, dentists

## Abstract

Little is known about the extent to which dentists have implemented COVID-19 infection control guidelines and the factors influencing this process in daily practice. This national online survey assessed the implementation of enhanced infection control guidelines in daily practice, and explored dentist related factors influencing their application, more specifically dentist infection status and their perceived risk of cross-infection in the dental setting. The survey was validated, pretested and carried out in 2020. A total of 1436 dentists participated, of whom 9.1% presumably had COVID-19 infection experience. At least 75% of dentists complied with the core part of the recommended protective measures protocol. For each patient treated during the pandemic, an additional cost of 10–30 EUR (86.7%) and an extra time of 10–30 min (70.7%) was estimated. A stepwise binary logistic regression analysis revealed that dentists assumed to have experienced COVID-19 reported a higher self-perceived risk of virus acquisition (β = 2.090; *p* = 0.011), lower concern of getting infected (β = 0.576; *p* = 0.027), and lower confidence in being able to prevent disease transmission in the dental setting (β = 0.535; *p* = 0.022). Some parts of the protective measures were more difficult to apply than others; however, there was no indication of increased disease acquisition in the dental setting.

## 1. Introduction

More than a year has passed since the outbreak of the COVID-19 pandemic, with an increasing number of people worldwide being infected by the SARS-CoV-2 virus and its variants identified in, e.g., the United Kingdom, South Africa, Brazil, United States of America, and India [1,2,3]. In spite of introducing protective measures and vaccination programs for the population, new emerging variants of the virus mean that countries are still at risk, even when, momentarily, they seem to have the infection under control. Thus, human-to-human transmission continues to occur through airborne droplets, direct mucous contact, or contact with contaminated surfaces [4,5].

As health professionals, dentists are highly exposed to airborne droplets and close patient contact while providing dental treatment. In this context, dentists are considered at high risk of getting infected with coronaviruses acquired from patients, and for transmitting them via cross-infection in the dental setting [6,7,8].

The World Health Organisation (WHO), together with national governments around the world, issued guidelines regarding precautionary measures to be adopted by dentists while treating patients as part of the initiatives to control the pandemic [9,10,11]. Few national studies have investigated the level of infection among dentists, which, in its turn, is associated with dentists’ knowledge, attitudes, perceptions, and awareness of viral transmission in daily practice [12,13,14]. A recent study including data from 36 countries showed that the reported routine use of FFP2/N95 masks was the only measure significantly associated with a reduced risk of viral infection acquisition by dentists [15].

In Belgium, oral health care is essentially provided by private practitioners who work individually or in group practices. Their individual implementation of enhanced international and national infection control guidelines affects the course of the pandemic. To date, little is known about the extent to which they have implemented these guidelines and the factors influencing this process in daily practice. This knowledge is considered of broad interest for providing a basis for guidelines to be developed or fine-tuned in case of further viral epidemics.

The aims of the present survey were twofold: (1) to assess the implementation of enhanced international and national infection control guidelines in daily practice, and (2) to explore dentist related factors influencing their implementation, more specifically dentist infection status and their perceived risk of cross-infection in the dental setting.

## 2. Materials and Methods

### 2.1. Ethics, Study Design, and Sample

The study protocol was submitted to the Ethics Committee of UCLouvain, Belgium, and approved under the Belgian register number B403. The study was designed as a national cross-sectional online survey, which was carried out in two waves—a first one between 30 July and 9 August 2020, and a second from 18 August to 2 September 2020. The present survey is reported according to STROBE guidelines for cross-sectional studies [16]. Dentists were asked to accept or to decline the invitation by either ticking a box declaring that they had read the privacy policy and voluntarily approved data collection and processing, or a refusal box, automatically closing the questionnaire. In Belgium, there were 10,080 dentists registered either as general dentists or specialists at the time of this survey [17]. A convenience sample of dentists registered in the contact lists of the main dental associations in Belgium (*n* = 8290) was invited by e-mail to participate in the survey. Three reminders were sent to potential participants by the associations. It was anticipated to include at least 10% of practitioners, representing both the Dutch and French language communities.

### 2.2. Participants

Participants in this survey were general dentists and officially registered specialists in the field of periodontics, orthodontics, and maxillofacial surgery working either in private practice, hospital settings, or in administration. All dentists were included in the descriptive analysis, while only general dentists were considered for further analyses.

### 2.3. Development and Validation of the Online Survey

The electronic questionnaire used in the present study, originally developed in English, was validated in an earlier project and consisted of four domains: (1) personal information, (2) symptoms or signs related to COVID-19, (3) working conditions and personal protection equipment (PPE) implemented during the COVID-19 outbreak, and (4) self-perceived knowledge and risk of virus transmission [12,18]. The original survey was complemented with questions related to the prescription of pain-killers, anti-inflammatory drugs, and antibiotics, the estimated extra cost and time spent on dental treatment of individual patients during the pandemic, and the usefulness of the information provided by governmental agencies and dental associations. The complete survey is described in Table S1 (Supplementary Material).

The survey was initially translated from the original English version into French and Dutch by two researchers competent in public health dentistry from each language group and with a very good knowledge of English to ensure its conceptual equivalence. Next, the French and Dutch versions were back-translated into English by two translators from each language group not belonging to the group of researchers. Subsequently, semantic adjustments were made to both versions, which were then reviewed by nine dentists for their comprehensibility. Final semantic adjustments were made following dentists’ comments and remarks.

To determine the reliability of the French and the Dutch versions of the survey, they were tested–retested with an interval of 1 week by 29 dentists. The intraclass correlation coefficient (ICC) of the test–retest was 0.84 (95% CI: 0.83–0.85). The electronic survey used the Qualtrics^xm^ (Qualtrix, Seattle, WA, USA) platform, and all results were anonymized. The participants were invited by email via the dental associations, presenting links to both language versions of the survey.

### 2.4. Missing Data

The dentists who completed the survey answered all questions, as the Qualtrics^xm^ platform was programmed in a way that did not allow empty answers. However, dentists could decline their participation at any moment, being informed that the data collected up to that moment were registered for analysis. The number of missing answers was calculated and reported for individual variables.

### 2.5. Statistical Analysis

Replies to the survey were exported in an Excel spreadsheet (Office 365 package, Microsoft, Redmond, WA, USA), and data were checked for consistency and, further, transferred to SPSS ™ (IBM SPSS 26.0.0.0 64-bit, IBM, Armonk, NY, USA) for statistical analysis. The demographic characteristics of the participants who completed the survey were compared with those who only partially completed it. Descriptive statistics in terms of absolute and relative frequency were used to present the characteristics of the sample. Further analyses, restricted to general dentists, were performed after weighting the data in accordance with population data in order to correct for sampling and participation bias. These analyses considered the representativeness of participating dentists by geographic location, gender, and age according to available data on workforce characteristics [17].

In some cases, answers were aggregated in order to obtain dichotomized data (Table S1). The variables related to protective measures were grouped as follows: appointment organization, waiting area organization, surface cleansing, mouth rinsing protocol, aerosol control (mask, gown, gloves, limiting use of rotating instruments, etc.) and hand hygiene procedures. Comparisons between proportions were tested using the χ2 test for dichotomous variables, while those between two paired groups were tested using the Wilcoxon test at a significance level of 5%. Stepwise binary logistic regression analyses were performed on categorized data to identify variables associated with the main outcome, i.e., the number of dentists infected with COVID-19. The dentists were considered infected if they reported having had the disease, having being hospitalized/tested positive for COVID-19, or having presented at least one major symptom (cough, difficult breathing, smell or taste loss) or two minor symptoms (headache, diarrhea, sore throat, nasal congestion, pain, fatigue) associated with the disease according to WHO [19] and SCIENSANO [20].

### 2.6. Confidentiality and Data Retention

Identification of the participants was not possible, and their responses were anonymous and confidential. Due to privacy reasons, age was recorded in decades (Table 1) and place of professional activity at district level in order to prevent back-tracing of individual respondents. Recorded data were stored on the server of the survey platform according to their data safety protocol (https://www.qualtrics.com/platform/security/, accessed on 26 July 2021). Access was restricted to one of the authors (PB) via password.

## 3. Results

A total of 1436 dentists participated in the survey, of whom 1084 (75.5%) completed the whole questionnaire while 352 provided partial answers. Table 1 describes the main characteristics of the sample. Female dentists were more numerous (58.8%) than their male counterparts. A total of 1322 general dentists and 80 specialists replied to the survey, while occupational information status was missing for 34 respondents. The overall participation rate was 14.1–11.7% for Brussels-capital region, 15.3% for Flanders, and 11.8% for Wallonia.

The comparison of the characteristics of participants who completed the questionnaire with those who only partially completed it showed no significant differences regarding age groups (χ2 test, *p* = 0.07), gender (*p* > 0.05), professional activity (*p* = 0.16), or type of practice (*p* = 0.39), while the regions of Brussels and Wallonia were overrepresented (*p* < 0.001).

The sample correlated rather well to the population characteristics of Belgian dentists (r^2^ = 0.7, *p* < 0.01) when taking into account age, gender, and region. Using weighting (coefficients ranging from 0.51 to 3.31, mean of 1.15 ± 0.58), a perfect correlation could be obtained. The sample was also well distributed geographically up to district level (r^2^ = 0.88 without weighting and r^2^ = 0.91 after weighting, *p* < 0.001).

**Figure 1:**
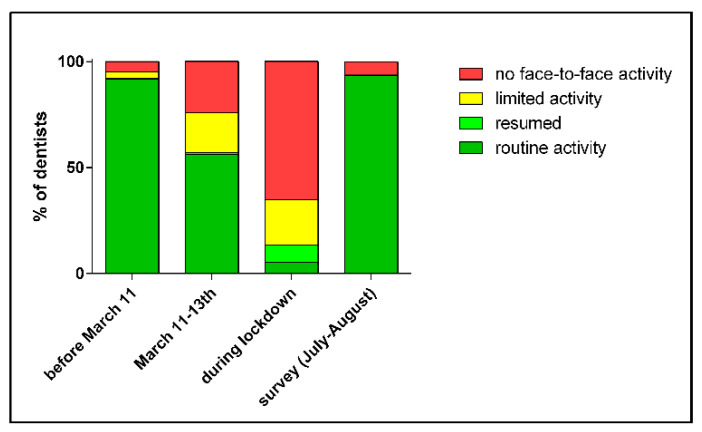
Reported clinical activity of dentists at the time that the WHO declared the COVID-19 pandemic (11 March), during the lockdown period in Belgium from 14 March 2020 (start of first lockdown) to 4 May 2020 (end of strict confinement), and at the time of the survey.

Figure 1 presents the reported clinical activity of dentists at the time that the WHO declared the COVID-19 pandemic (11 March), during the lockdown period from 14 March 2020 (start of first lockdown) to 4 May 2020 (end of strict confinement), and at the time of the survey. During the lockdown period, clinical activity dropped dramatically, with most practitioners limiting activity to emergency services or remote advice. After the end of the strict confinement, at the moment of the survey, most practitioners had resumed face-to-face clinical activity (94.7%) while a remaining minority only remotely advised patients (0.9%) or did not open their dental office to patients (4.4%).

The majority of dentists adopted the international and national recommended infection control guidelines regarding personal protection, particularly the wearing of FFP2/N95 masks, gloves, and protective glasses/visors (>75%). A total of 82.2% of the dentists reported using FFP2/FFP3 masks, while 16.9% used surgical masks and 0.9% used no masks. The routine use of FFP2/FFP3 masks, considered internationally and nationally to be of utmost importance to prevent airborne infection, was reported by 74.3% of dentists, while 19.4% used them only for aerosol generating procedures.

In addition, the protection of patients and prevention of cross-contamination by limiting attendance to emergency care and checking patient’s general health status, together with asking the patient to rinse the oral cavity with 1% H_2_O_2_ or 1% iodine polyvidone to reduce the SARS-CoV-2 salivary load, were frequently adopted (74%). The same pattern was observed in relation to ventilation of the dental office and surface cleansing protocols (>70%). The least frequently applied guidelines were maintaining a distance of 1.5 m between patients in the waiting room (55.8%) and checking patient’s body temperature (36.7%). Only 40% of the respondents reported reducing the use of high speed/ultrasonic instruments or routinely applying rubber dam during operative procedures.

Table 2 shows the implementation of the different parts of the protective measures protocol based on aggregated variables, as described in the Materials and Methods section. Surface cleansing instructions and hand hygiene procedures were applied by most dentists (86.2% and 82.5, respectively). This was also the case for the mouth rinse protocol which was applied by 74.6% of the respondents. Only 7.9% reported applying all recommended protective measures. Dentists aged 55 years or above less frequently implemented guidelines related to appointment organization (45.3% vs. 54.7%), aerosol control (27.3% vs. 41.1%), and mouth rinse protocol (71.3% vs. 78.8%) than younger ones (χ2, *p* < 0.001).

The dentists reported that they considered themselves well-informed about COVID-19 (76.0%). Whereas a majority of practitioners felt well-informed by professional associations (64.7%), only 23.1% reported this for governmental authorities (Wilcoxon test, *p* < 0.001).

Almost two thirds of the dentists (60.9%) considered their self-perceived risk of virus acquisition in the dental setting high, and 50.6% reported being highly concerned about it. Only 36.8% of dentists felt confident about being able to prevent infection transmission in the dental setting.

In total, 9.1% of the dentists presumably had COVID-19 infection experience, 25 dentists reported having had a COVID-19 infection confirmed by test, of which two had been hospitalized, and another 94 reported having had one major symptom (cough, difficult breathing, smell or taste loss) or two minor symptoms (headache, diarrhea, sore throat, nasal congestion, pain, fatigue) associated with COVID-19.

According to the respondents, the implementation of the recommended infection control guidelines generated an estimated additional cost from 10 to 30 EUR (86.7%) and an additional time spent from 10 to 30 min (70.7%) for the treatment of each patient during the pandemic.

During lockdown, an increased prescription rate of antibiotics and anti-inflammatory drugs/analgesics was reported by 18.3% and 23.0% of the practitioners, respectively. The majority of dentists (>85%) prescribed medicines as usual or when indicated by the clinical condition.

Stepwise binary logistic regression analyses were run with dentists presumed having experienced COVID-19 infection as dependent variable (Table 3). The first analysis tested the association with the implementation of the international and national recommended infection control guidelines. The implementation of the cleansing protocol and age groups 55–64 years and >65 years were independent variables significantly associated with lower COVID-19 infection experience.

The second analysis (Table 4) tested the association with dentists’ self-perceived risk of virus acquisition, as well as dentists’ concern/confidence of preventing transmission in the dental setting. Dentists having experienced COVID-19 reported a high self-perceived risk of virus acquisition, lower concern of getting infected, and lower confidence in being able to prevent disease transmission in the dental setting.

## 4. Discussion

The main finding of this survey was that at least 75% of dentists complied with the core part of the recommended protective measures protocol, i.e., hand hygiene procedures, mouth rinse protocol, and surface cleansing protocol. Some parts of the protective measures protocol were clearly more difficult to apply; specifically, appointment organization, waiting area organization, and aerosol control. For obvious reasons, measures limiting the use of rotating instruments were difficult to comply with (40%), due to necessary interventions in oral health care delivery. Only a small proportion of dentists (7.9%) implemented all recommended international and national enhanced infection control guidelines.

Even though the infection control measures were only implemented to a certain extent, they seem to have been sufficient to maintain the rate of dentists experiencing COVID-19 infection comparable to that of the general population and to that of other healthcare workers in Belgium [20]. The proportion of Belgian dentists who reported at least one major or two minor symptoms COVID-19 related symptoms (8.0%) was comparable to that reported by Wolf et al. [21] in Switzerland and Liechtenstein (10.3%), but was lower than the observations made by Persoon et al. [22] in the Netherlands for dentists reporting at least one COVID-19 symptom (19.2%) and by Estrich et al. [14] in USA for dentists reporting symptoms (17.8%).

One may question the uncertainty associated with assessing the true rate of infection among dentists due to the presence of asymptomatic SARS-COV-2 infection [8], the sensitivity as well as the specificity of various testing systems [23], and, simply, due to the limited availability of testing systems at the time of the survey. While we acknowledge these uncertainties, we consider that they were equally present when estimating COVID-19 infection rates in the general population and in other healthcare workers, which makes their comparison acceptable. Furthermore, the incidence rate of COVID-19 infection among dentists over 6 months seems to be very low (2.6%), as recently documented by Araujo et al. [24]. Compliance with the protective measures protocol was also shown to prevent virus transmission to patients [25].

In this study, the participation rate of 14.1% is in line with that of some surveys dealing with COVID-19 infection among dentists [13,15] but lower than other publications in recent literature [14,24,26]. The fact that the survey was launched during summer 2020 when the majority of the dentists had resumed their activities, thus being very busy with patient treatments and the fact that the survey was carried out in the holiday period, might have influenced the rate of participation. In this context, an important aspect to highlight is that dentists participating in this survey were shown to be representative of the general population of dentists in Belgium [17,27].

The potential acquisition and transmission of the SARS-CoV-2 virus in the dental setting [7,27,28], particularly during aerosol generating dental procedures [6,28,29,30,31], elicits questions about adherence to recommended protective measures. Due to the potential risk of transmitting the SARS-CoV-2 virus in the dental setting, we analyzed dentists’ adherence to protective measures, dentist related factors associated with the implementation of protective measures and their link with presumed positivity rate of dentists as well as their perceived risk of infection transmission.

On one hand, dentists aged 55 years or older, dentists implementing the cleansing protocol, dentists who were less concerned about getting infected, or those with lower confidence in being able to prevent virus acquisition in the dental setting were significantly less likely to have experienced COVID-19 infection. Since a considerable number of dentists aged 55 years or older did not resume their activities at the time of the survey, it might partially explain their low reported experience with COVID-19 infection. On the other hand, dentists with a high self-perceived risk of virus acquisition were significantly more likely to have experienced COVID-19 infection. Over the years, dentists have implemented a series of protective measures against other transmissible diseases while treating patients in the dental setting. For example, dentists have been wearing masks, gloves and protective glasses for routine oral health care delivery for many years. The addition of an enhanced cleansing protocol in response to the ongoing pandemic was a significant factor for limiting the acquisition of the SARS-CoV2 virus. Together, these findings indicate that the risk of transmission of the SARS-CoV-2 virus in daily practice can be managed, although some authors reported a considerable risk of getting infected in the dental setting [6,32,33,34].

Although few studies reported on their dental treatment needs, it is important to bear in mind that protective measures should be applied not only for presumed COVID-19 patients but also for post-COVID-19 patients with or without prolonged symptoms [35].

In an earlier study, Schwendicke et al. [36] analyzed the economic impact of the SARS-CoV2 virus on dental practices and reported that the longer the Covid-19 mitigation measures are maintained, the greater the financial consequences for dental practices, particularly for those with higher operational costs. The analysis of the economic impact of the pandemic on daily practice was further developed in the present study with general dentists estimating that each patient contact generated an additional cost between 10 and 30 EUR and required an additional time of 10–30 min for the implementation of recommended infection control guidelines. In Poland, a survey covering the period from May to October 2020, 70.7% of dentists reported a treatment price increase, while 68.1% experienced an increase in time spent admitting each patient [37]. These findings are relevant for health care authorities considering financial compensation for dentists when enhanced infection control policies need to be implemented. In Belgium, some financial compensation for dentists was granted by the National Institute for Health and Disability Insurance [27].

In our study, limiting the access to dental care during the lockdown period led to an 18% and 23% increase in antibiotics and anti-inflammatory drugs/analgesics prescription by dentists. In England, studies assessing the management of dental pain by analgesics and dental infection by antibiotics as adjuncts to local treatment during the lockdown period showed similar trends, with an increase in antibiotics prescription ranging from 22% to 25%, while the finding for analgesics prescriptions of 84% largely exceeded our figures [38,39]. To combat further development of antibiotic resistance, the appropriate prescription of antibiotics as adjunct to local treatment of oral infections is of utmost importance [40,41]. During the lockdown period, 85% of Belgian dentists reported that their antibiotics prescription practice was justified by the clinical condition of the patients.

During pandemics, early and clear communication between governments and health professionals is essential [1,2,10,11]. In this survey, dentists reported that, initially, they were mainly informed by a collaborative initiative between universities and dental associations, while governmental agencies were mainly focusing on acute general medical care and the situation in nursing homes. This is reflected in their reported satisfaction with obtained information; 23.1% were satisfied with governmental communication and 64.7% with information provided by dental associations.

Extrapolation of the findings of the present study has limitations due to its relatively small sample size. However, weighting of the data was undertaken to correct for possible selection bias, allowing overall conclusions to be drawn for the situation of Belgian dentists. Furthermore, the cross-sectional design of this survey allows only the establishment of associations between the dependent variable reported COVID-19 experience and a set of independent variables related to dentists and their way of coping with the pandemic. By design, this survey was addressed to dentists, and no questions were included about the use of protective measures by other dental team members. It would, thus, be of interest to investigate the impact of COVID-19 on the whole dental team (auxiliary clinical and administrative staff, dental technicians). Finally, a questionnaire survey relies on the memory of the participants, which might not be as accurate as would be desirable, a problem inherent to this type of research.

## 5. Conclusions

Several factors impacted the implementation of the recommended protective measures. Some parts of the protective measures protocol were clearly more difficult to apply. However, there was no indication of an increased disease transmission in the dental setting. This information can be used for further refinement of the protective measures protocol. In addition, this study underlines the need for strategies to bring together relevant expertise in case of disease outbreaks and of effective communication with dental professionals.

## Figures and Tables

**Table 1:** Characteristics of the dentists who participated in the survey (*n* = 1436).

Variables	Categories	Number (%)
Region (missing = 1)	Brussels	205 (14.3%)
	Flanders	775 (54.0%)
	Wallonia	455 (31.7%)
Age group (years)	≤34	207 (14.4%)
	35–44	154 (10.7%)
	45–54	274 (19.1%)
	55–64	516 (35.9%)
	≥65	285 (19.8%)
Gender	Female	845 (58.8%)
	Male	591 (41.2%)
Language spoken (missing = 1)	Dutch	765 (53.3%)
	French	670 (46.7%)
Professional activity (Missing = 34)	General dentist	1322 (94.3%)
	Orthodontist	46 (3.3%)
	Periodontist	29 (2.0%)
	Maxillofacial surgeon	5 (0.4%)
Practice type (missing = 34)	Private owner	918 (65.5%)
	Private coworker	409 (29.2%)
	Hospital practice	36 (2.5%)
	Other	39 (2.8%)
Reported infection rate *	Brussels	55 (0.04%)
	Flanders	25 (0.02%)
	Wallonia	46 (0.03%)
Working status at the moment of the survey (missing = 54)	None	61 (4.4%)
	Yes, remote advice	12 (0.9%)
	Yes, face to face	1309 (94.7%)

* Infection was defined as having had the disease, being hospitalized, testing positive, or reporting at least one important symptom or two minor symptoms according to international and national guidelines.

**Table 2:** Implementation (number of dentists, percentage) of the different parts of the protective measures protocol by dentists based on aggregated variables.

Protective Measure	Number	%
Appointment organization	537	49.5
Waiting area organization	422	38.9
Surface cleansing	934	86.2
Hand hygiene procedures	894	82.5
Mouth rinse protocol	809	74.6
Aerosol control (mask, visor, gown, rubber dam, and limiting rotating instruments)	401	33.5
All measures	86	7.9

**Table 3:** Stepwise binary logistic regression of variables associated with COVID-19 infection experience.

Variables	Dentists Having Experienced COVID-19 Infection		
	β	SE	*p* Value
Age group 55–64 years	0.149	0.286	<0.0001
Age > 65 years	0.522	0.492	0.023
Implementation of cleansing protocol	0.030	0.310	0.003

**Table 4:** Stepwise binary logistic regression of variables associated with COVID-19 infection experience.

Variables	Dentists Having Experienced COVID-19 Infection		
	β	SE	*p* Value
Dentists with high self-perceived risk of virus acquisition in the dental setting	2.090	0.291	0.011
Dentists with low concern of getting infected in the dental setting	0.576	0.251	0.027
Dentists with low confidence of being able to prevent transmission in the dental setting	0.535	0.273	0.022

## Data Availability

The data presented in this study are openly available in [FigShare] at [doi:10.6084/m9.figshare.14945814, accessed on 10 July 2021].

## Supplementary Materials

The following is available online at https://www.mdpi.com/article/10.3390/ijerph18168381/s1, Table S1: Description of the questionnaire and aggregation of derived variables.
