# Supplementary material for: Dentist Related Factors Associated with Implementation of COVID-19 Protective Measures: A National Survey
Source: Int J Environ Res Public Health. 2021 Aug 8;18(16):8381. doi: 10.3390/ijerph18168381 (PMC8391182; doi:10.3390/ijerph18168381)
Supplement: Supplementary file 1 [file ijerph-18-08381-s001.zip › ijerph-1315080-supplementary.pdf]

International Journal of
Environmental Research and Public Health

MDPI

Supplementary Material

Dentist Related Factors Associated with Implementation of COVID-19 Protective Measures: A National Survey

Table S1. Description of the questionnaire and aggregation of derived variables.

Question	Answers options	Aggregation/dichotomization, derived variables
Demographics:<br>Age group according to WHO<br>Gender<br>Region<br>District	 < 24 yr; 25-34 yr; 35-44 yr; 45-54 yr; 55-64 yr; 65-74 yr ; >75 yr<br>Female/male/other<br>Brussels, Wallonia, Flanders<br>Lock down menu with all districts listed	 Age groups <24 and 25-34 years as well as 64-75 and >75 were aggregated
Reason for not answering this questionnaire:	Not applied (continue the survey)<br>Refusal<br>• No interest<br>• No time<br>• Age related reason<br>• Health reasons	
Please describe your current dental practice workplace:	Private practice (owner/co-owner)<br>Public sector<br>Both public and private (salaried)<br>Academic/research<br>Non-clinical/administration<br>Hospital practice	Public sector, academic/research , non-clinical/ administration were aggregated to “other”.
Are you a:	General dentist<br>Specialist in orthodontics<br>Specialist in Periodontics<br>Specialist in maxillo-facial surgery	
Since COVID-19 emerged, you have:	No symptoms<br>Tested positive for Covid<br>Hospitalized<br>Had one or more symptoms	The dentists were considered infected if they reported having had the disease, having being hospitalized/tested positive for COVID-19 or having presented at least one major symptom (cough, difficult

	• Fever (>37,3° C)<br>• Cough<br>• Fatigue<br>• Difficulty breathing<br>• Nasal congestion<br>• Headache Nose bleeds<br>• Sore throat<br>• Diarrhoea<br>• Loss of smell<br>• Loss of taste<br>• Conjunctivitis	breathing, smell or taste loss) or two minor symptoms (headache, diarrhoea, sore throat, nasal congestion, pain, fatigue).
Are you currently working as a dental care provider?	Yes, seeing/treating patients face to face<br>Yes, remotely providing advice/triage only<br>No	
Did you apply the special fee code to your patients for phone consultations?	Yes, for all patients<br>Yes, for some patients<br>No	
From March 14th 2020* to May 4th **, which of the following describes your work:<br>(*Belgium went in lockdown; ** end of strict confinement)	You kept working clinically as usual<br>You limited your face-to-face clinical activity to selective/urgent care<br>You stopped all face-to-face activities<br>You stopped all face-to-face activities and provided remote advice/triage	
If you have limited your face-to-face clinical activity to urgent care, when did you start limiting?	Before March 11th<br>Between March 11th and March 14th<br>From March 15th<br>Sometime during the lockdown<br>Did not limited face-to-face activities	
If you have stopped your face-to-face clinical activity, when was that?	Before March 11th<br>Between March 11th and March 14th<br>From March 15th<br>Sometime during the lockdown<br>Did not limited face-to-face activities	

If you continued seeing patients face-to-face after March 14th, 2020, which of the following precautionary measures have you taken?	No additional measures<br>Phone triage<br>Appointments reduces as to not fill the waiting room<br>Postponement of appointment to elderly or vulnerable people<br>Verify the patient's current health status on/before arrival<br>Detecting the patient's body temperature	Aggregated to variable 'appointment organisation':<br>Yes: >3 positive answers.
	Handle disinfection several times a day<br>Disinfection of push buttons, chairs, several times a day<br>Surface disinfection with 70% ethyl alcohol<br>Surface disinfection with other (please state what)………<br>Disinfection of surfaces with 0.5% sodium hypochlorite<br>Usual disinfectant with other active ingredients (please state what)………	Aggregate to variable 'surface cleansing'<br>Yes: cleansing of handles OR buttons AND surfaces (any product mentioned).
	Space of at least one and half metre between patients in the waiting room<br>Patients wait outside the practice until called into the clinic for their appointment<br>Mask for the patient whilst in the waiting room<br>Frequent ventilation of waiting rooms<br>Limiting access to toilets<br>Removal of magazines and books from the waiting area<br>Storage of coats, bags and other items outside the operating area<br>Sanitizing patients' hands	Aggregated to 'waiting area organisation'<br>Yes: 4 positive answers on sanitizing patients' hands AND mask for the patient AND space of at least one and half metre between patients AND semoval of magazines and books AND ≥ 2 patients wait outside the practice OR<br>Limiting access to toilets OR storage of coats, bags and other items OR frequent ventilation of waiting rooms.
	Pre-operative rinse with mouthwash containing 1% hydrogen peroxide<br>Pre-operative rinse with mouthwash containing chlorhexidine 0.12-0.2%<br>Pre-operative rinse with mouthwash containing 0.2-1% iodopovidone<br>Pre-operative rinse with mouthwash containing alcohol and essential oils<br>Pre-operative rinse with mouthwash containing 0.05-0.10% Cetylpyridinium chloride<br>Rinse with diluted mouthwash	Aggregated to variable 'mouth rinse protocol':<br>Yes: positive answer to 1% H2O2 OR 1% iodopovidone.
	Limiting the use of high speed rotative instruments and ultrasonic devices<br>Limiting the use of 3 function syringe<br>Refrain from using the spittoon<br>Disposal of all disposable protective devices and disinfection of non-disposable devices	Part of aggregated variable 'aerosol control':<br>Yes: limiting the use of high speed rotative instruments and ultrasonic devices AND limiting the use of 3 function syringe AND refrain from using the spittoon.

	Washing operators' hands before and after each procedure	Part of aggregated variable 'hand hygiene procedures' Yes: Washing operators' hands before and after each procedure AND routinely wearing any type of gloves.
When seeing patients, which of the following protective equipment have you adopted for your use?	Not used/Routine use/Used only in aerosol-generating procedures<br>• Surgical mask<br>• FFP2/N95 mask<br>• FFP3 mask<br>• Hood<br>• Disposable visor<br>• Sterile microfiber disposable gown<br>• Water-repellent TNT disposable gown<br>• Another disposable gown<br>• Non-disposable/reusable gown<br>• Safety glasses or visor<br>• Sterile disposable gloves<br>• Non-sterile disposable gloves<br>• Rotating instrument with anti-retraction valve<br>• Rubber dam	Part of aggregated variable 'aerosol control':<br>Yes: > 4 positive answers to routinely wearing FFP2/N95/FFP3 AND any type of gown AND hood AND safety glass OR visor AND rubber dam AND limiting the use of high speed rotative instruments OR ultrasonic devices AND limiting the use of 3 function syringe AND refrain from using the spittoon.
Do you have your patients tested for COVID19 before dental treatment?	Yes<br>No	
Have you been tested for COVID-19?	Yes<br>No	
Have you completed an educational course on COVID-19?	Yes<br>No	
Do you think that you know enough about COVID-19?	Yes<br>No	

Do you believe that catching COVID-19 at work is a real risk for dentists?	4-point Likert scale:<br>Very unlikely/unlikely/likely/very likely	Aggregated to very unlikely and unlikely versus likely and very likely
How concerned are you about the risk of catching COVID19 at Work?	4-point Likert scale:<br>Not concerned at all/reasonably concerned/somewhat concerned/very concerned	Aggregated to not concerned at all and reasonably concerned versus somewhat concerned and very concerned
How sure are you that you can avoid becoming infected with COVID-19 during work?	4-point Likert scale:<br>Not confident at all/reasonably confident/somewhat confident/very confident	Aggregated to not confident at all and reasonably confident versus somewhat confident and very confident
Do you believe the risk of coronavirus transmission in the dental practice is:	Less than the risk associated with going to a supermarket<br>Comparable to the risk associated with going to a supermarket<br>Higher than the risk associated with going to a supermarket	
If you have continued to work as a dentist, has your antibiotic prescribing practice changed during the pandemic?	No<br>Yes, more prescriptions have been issued<br>Yes, less prescriptions have been issued<br>I am not working as a dentist	Aggregated to more prescriptions versus other choices.
If you have prescribed more antibiotics than usual, what has influenced this?	Clinical decision that antibiotics were appropriate<br>Antibiotics unlikely to help but felt I needed to offer something other than just advice<br>Demand from patients<br>Purely preventive since it may be some time before patient can access dental care<br>Other (please add detail):	
If you have continued to work as a dentist, has your painkiller/anti-inflammatory prescribing practice changed during the pandemic?	No<br>Yes, more prescriptions have been issued<br>Yes, less prescriptions have been issued<br>I am not working as a dentist	Aggregated to more prescriptions versus other choices.
If you have prescribed more painkiller/anti-inflammatory than usual, what has influenced this?	Clinical decision that painkiller/anti-inflammatory were appropriate<br>painkiller/anti-inflammatory unlikely to help but felt I needed to offer something other than just advice<br>Demand from patients<br>Purely preventive since it may be some time before patient can access dental care<br>Other (please add detail):	
How do you think COVID-19 will change dental practice in the future?	4-point Likert scale: Not at all likely/Unlikely/Quite likely/Very likely<br>• Video consultations will become more common<br>• Remote triage prior to appointment	Aggregated to not at all likely and unlikely versus quite likely and very likely

	• Longer recall intervals<br>• Remote provision of preventive advice<br>• Increase in infection control requirements<br>• Increase in Personnel Protective Equipment requirements<br>• Increased costs for patients<br>• Increased costs for dentist<br>• No change to the way dentistry is practiced	
Was the information provided to you on COVID-19 by the Belgian health authorities:	Not sufficient<br>Reasonably sufficient<br>Somewhat sufficient<br>Entirely sufficient	Aggregated to not sufficient AND reasonable sufficient versus somewhat sufficient AND entirely sufficient.
Was the information provided to you on COVID-19 by the Belgian dental associations:	Not sufficient<br>Reasonably sufficient<br>Somewhat sufficient<br>Entirely sufficient	Aggregated to not sufficient AND reasonable sufficient versus somewhat sufficient AND entirely sufficient.
Do you feel that your patients refrain from dental care:	Not at all<br>Only non-urgent care, by a small number of patients<br>Only non-urgent care, by a large number of patients<br>Necessary care, by a small number of patients<br>Necessary care, by a large number of patients	
What do you think the extra cost is of safety measures required by COVID19 for treating patients in your dental practice?	0 €<br><10 €<br>11-20 €<br>21-30 €<br>31-40 €<br>>40 €	
What do you think the extra time is for safety measures required by COVID19 for treating patients in your dental practice?	0 min<br><10 min<br>11-20 min<br>21-30 min<br>31-40 min<br>>40 min	
